# Proton pump inhibitor chemosensitization in human osteosarcoma: from the bench to the patients’ bed

**DOI:** 10.1186/1479-5876-11-268

**Published:** 2013-10-24

**Authors:** Stefano Ferrari, Francesca Perut, Franca Fagioli, Adalberto Brach Del Prever, Cristina Meazza, Antonina Parafioriti, Piero Picci, Marco Gambarotti, Sofia Avnet, Nicola Baldini, Stefano Fais

**Affiliations:** 1 Chemotherapy Unit, Istituto Ortopedico Rizzoli, Bologna, Italy; 2 Orthopaedic Pathophysiology and Regenerative Medicine Laboratory, Istituto Ortopedico Rizzoli, Bologna, Italy; 3 Pediatric Oncology Unit, OIRM Torino, Torino, Italy; 4 Pediatric Oncology Unit, Istituto Nazionale Tumori Milano, Milano, Italy; 5 Pathology Department, Istituto Gaetano Pini, Milano, Italy; 6 Experimental Oncology Laboratory, Istituto Ortopedico Rizzoli, Bologna, Italy; 7 Pathology Unit, Istituto Ortopedico Rizzoli, Bologna, Italy; 8 Anti-Tumour Drugs Section Department of Therapeutic Research and Medicines Evaluation, Istituto Superiore di Sanità, Rome, Italy

## Abstract

**Background:**

Major goals in translational oncology are to reduce systemic toxicity of current anticancer strategies and improve effectiveness. An extremely efficient cancer cell mechanism to avoid and/or reduce the effects of highly cytotoxic drugs is the establishment of an acidic microenvironment, an hallmark of all malignant tumors. The H + −rich milieu that anticancer drugs meet once they get inside the tumor leads to their protonation and neutralization, therefore hindering their access into tumor cells. We have previously shown that proton pump inhibitors (PPI) may efficiently counterattack this tumor advantage leading to a consistent chemosensitization of tumors. In this study, we investigated the effects of PPI in chemosensitizing osteosarcoma.

**Method:**

MG-63 and Saos-2 cell lines were used as human osteosarcoma models. Cell proliferation after pretreatment with PPI and subsequent treatment with cisplatin was evaluated by using erythrosin B dye vital staining. Tumour growth was evaluated in xenograft treated with cisplatin after PPI pretreatment. Subsequently, a multi-centre historically controlled trial, was performed to evaluate the activity of a pre-treatment administration of PPIs as chemosensitizers during neoadjuvant chemotherapy based on methotrexate, cisplatin, and adriamycin.

**Results:**

Preclinical experiments showed that PPI sensitize both human osteosarcoma cell lines and xenografts to cisplatin. A clinical study subsequently showed that pretreatment with PPI drug esomeprazole leads to an increase in the local effect of chemotherapy, as expressed by percentage of tumor necrosis. This was particularly evident in chondroblastic osteosarcoma, an histological subtype that normally shows a poor histological response. Notably, no significant increase in toxicity was recorded in PPI treated patients.

**Conclusion:**

This study provides the first evidence that PPI may be beneficially added to standard regimens in combination to conventional chemotherapy.

## Introduction

Low pH is a major cause of tumor unresponsiveness to the vast majority of cytotoxic drugs mostly due to the fact the H + −rich tumor microenvironment leads to protonation of the drug causing both its neutralization outside the cells and abrogation of drug entry within the target cell [1]. The prime cause of tumor microenvironment acidification is the Warburg effect, which leads to overproduction of lactic acid by malignant tumors. However, this condition progressively selects cells that live in the acidic microenvironment due to overexpression and function of proton pumps that avoid intracellular acidification [2]. Vacuolar-type H + −ATPases play a key role in the acidification of tumor microenvironment [2]. Under experimental conditions, pretreatment of drug-resistant tumor cells with proton pump inhibitors (PPI) increases tumor cells sensitivity to a variety of anticancer drugs [3]. Pretreatment with PPI, followed by the administration of anticancer drug, both *in vitro* and *in vivo,* resulted in the most efficient approach. This effect was correlated with both an inhibition of ATPase activity and a PPI-induced marked increase in drug retention within tumor cells. PPI-mediated chemosensitization occured independently of both tumor histology and the type of cytotoxic drug [3].

More recently, it has been shown that the *in vivo* PPI-mediated effect on human tumors is transient, particularly in terms of pH gradient at the cellular level [4]. Lastly, PPI were able to increase sensitivity of cells responding to standard chemotherapy, but also to revert multidrug resistance (MDR) [3]. A PPI-based approach might therefore be extremely interesting to test both in solid tumors unresponsive to drugs and in tumors responding to chemotherapy but undergoing MDR and paying also the price of high level of toxicity of very aggressive chemotherapy. Osteosarcoma is a rare tumor with an overall incidence of 0.2 new cases/100,000. It is more frequently diagnosed in adolescents and young adults where it accounts for >10% of all solid cancers [5]. Currenty, strategy is based on a combination of surgery and chemotherapy. Chemotherapy is delivered before and after surgical removal of the tumor (neoadjuvant chemotherapy). The most effective drugs used for osteosarcoma are methotrexate (MTX), cisplatin (CDP), doxorubicin (ADM), and ifosfamide (IFO). In case of patients without evident metastatic disease at presentation the event-free survival is 60% at 5 years [6]. Late (>5 years) relapse are uncommon in patients with osteosarcoma (6,7). Inadequate surgical control of the tumor leads to local recurrence in about 5% of patients. In about 35% of patients tumor resistance towards the four-drug combination therapy is responsible for the failure of the systemic chemotherapy treatment [6]. The rationale for neoadjuvant chemotherapy is based on an early use of chemotherapy and on the possibility to assess chemosensitivity by means of histological evaluation of the chemotherapy-induced tumor necrosis on the surgical specimen [7]. There is a strong correlation between chemotherapy-induced tumor necrosis and prognosis in patients with osteosarcoma [8] with a higher probability of disease-free survival obtained in patients having a good histologic tumor response to neoadjuvant chemotherapy [6,7]. Hence, the histologic response to neoadjuvant chemotherapy is a very important predictive factor of survival and a reliable parameter of chemosensitivity. Previous studies have shown that the preoperative chemotherapy dose-intensification [9] or the preoperative deliver of intra-arterial cisplatin [10,11] are able to increase the percentage of cases with a good response to neoadjuvant chemotherapy. Both strategies are however associated with more severe side effects and discomfort for the patient [9,11]. Preclinical data showing that pretreatment of drug-resistant tumor cells with proton pump inhibitors (PPI), renders tumor cells more sensitive to a variety of anticancer drugs [3] have suggested that the administration of PPI as chemosensitizers might be an innovative approach to increase the sensitivity of osteosarcoma cancer cells to the currently used drugs. Moreover, an *in vivo* study performed in companion animals with spontaneous occurring tumors, including osteosarcoma, has shown an amazing clinical response to high dosage PPI/chemotherapy combination [12].

Based on the hypothesis that microenvironmental acidity may represent a key factor in tumor homeostasis, mostly involved in resistance to cytotoxic drugs, this study presents preclinical and clinical data evaluating the effectiveness of PPIs as chemosensitizer against human osteosarcoma. More specifically, this study was aimed at exploring the potential use of high dose PPI pretreatment in osteosarcoma patients undergoing neoadjuvant chemotherapy.

## Materials and methods

### In vitro studies

#### Cells

Continuous cell lines from human osteosarcoma (Saos-2, MG-63), obtained from the American Type Culture Collection (ATCC, Rockville, MD), were maintained in Iscove's Modified Dulbecco's Medium (IMDM, Invitrogen, Carlsbad, CA), supplemented with 10% fetal bovine serum (FBS, Mascia Brunelli, Milan, Italy), penicillin (100 U/ml) and streptomycin (100 mg/ml) (Invitrogen) at 37°C and 5% CO2. Only cells in exponential growth phase were used.

#### Cell proliferation assay

Cells were seeded in duplicate in 12-well plates (3x10^4^ cells/well for Saos-2 and 2x10^4^ cells/well for MG-63) in unbuffered medium. After 24 h the culture medium was replaced with fresh medium containing 60 μM esomeprazole (ESOM) (Sigma-Aldrich) dissolved in DMSO. As controls, cells were incubated with medium at the same concentration of DMSO. After 24 h the culture medium was replaced with fresh medium containing 0.5-5-50 μM cisplatin (Sigma-Aldrich). After 72 h, pH of the medium was tested and cells were harvested. The number of viable cells was evaluated by the erythrosin B dye vital staining [13]. Results were expressed as growth inhibition ratio in respect to cells in 0.5 μM cisplatin medium without ESOM pretreatment. The experiment was repeated three times in duplicate.

#### Statistical analysis

Statistical analysis was performed using the StatView™ 5.0.1 software for Windows (SAS Institute, Cary, NC). Differences were analysed using the non-parametric Wilcoxon Signed Rank test (significant level of p < 0.05).

### In vivo study

Female CB.17 SCID/SCID mice aged 4–5 weeks (Harlan, Italy) were kept under specific pathogen-free conditions and fed *ad libitum.* Mice were housed in micro-isolator cages, and all food, water, and bedding were autoclaved prior to use. Each mouse was injected subcutaneously in the right flank with 3 x10^6^ human osteosarcoma cells (Saos-2) that had been resuspended in 0.2 mL of RPMI-1640 containing 10% FCS. Once tumors became evident (at least 0.10 cm, approximately 10 days after the tumor cell injection), ESOM was administered by i.p. injection [4] at a dose of 25 mg/kg. Cisplatin was administered weekly by i.p. injection at a dose of 5 mg/kg. Tumor size (mm^3^) was measured three times per week with calipers with the formula length x width^2^. Morbidity was considered as the end-point according to standard clinical criteria including oversized tumor (>1 cm), weight loss (>20%), rough hair coat, and general illness. At least 5 mice were used for each treatment group. Data are expressed as the mean value of tumor weight with 95% confidence intervals. Mice were monitored for the duration of the *in vivo* experiments for body weight, hair ruffling, and the presence of diarrhea. Animal care was conformed to European Council Directive 86/609/EEC and the study was approved by institutional review board.

Differences between groups were analyzed by Mann-Whitney test, student T test or by ANOVA as appropriate. Data are expressed as mean 6 SD and p values reported are 2-sided.

### Clinical study

The study was a multi-centre historically controlled trial, evaluating the activity of a pre-treatment administration of PPIs as chemosensitizers in a neoadjuvant chemotherapy based on methotrexate (MTX), cisplatin (CDP), and adriamycin (ADM).

Patients aged ≤40 years with resectable nonmetastatic osteosarcoma of the extremities, with normal bone marrow, hepatic, cardiac and renal function, without contraindications to the use of MTX, CDP, ADM, were included into the study and after given written informed consent. Patients were instructed about the use of the study drug. In a separate sheet of the discharging letter they had to fill a table reporting the exact time of administration of the study drug and signs or symptoms related to the study drug.

**Figure 1:**
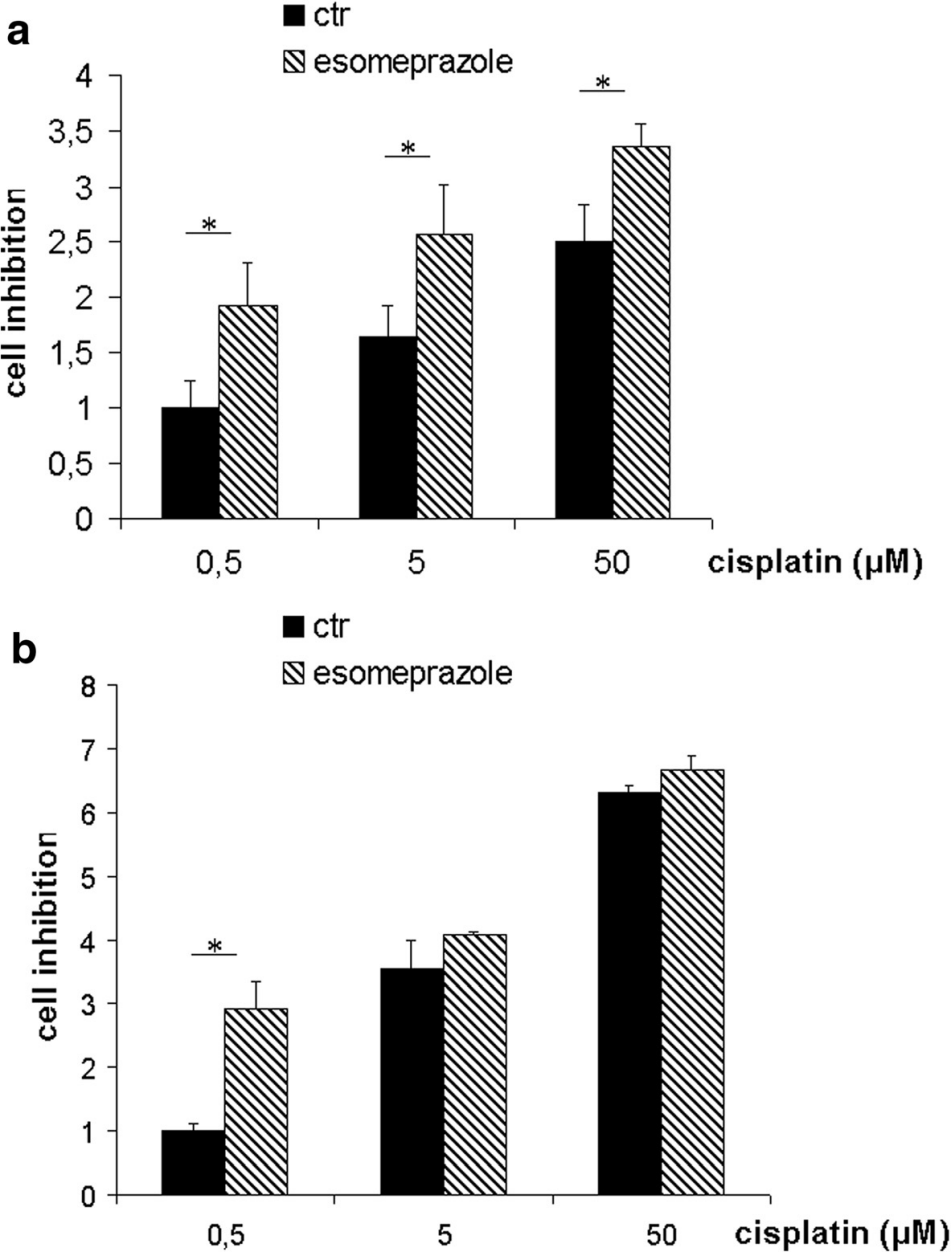
***In vitro *studies on PPI effect on osteosarcoma cells. (a)** Comparison of CDP activity with or without ESOM pre-treatment (60 μM) in MG-63 cells. The activity of CDP is significantly higher in pretreated cells (significant level * for p < 0.05) for all CDP doses. **(b)** Comparison of CDP activity with or without ESOM pre-treatment (60 μM) in Saos-2 cells The activity of ESOM is significantly higher in pretreated cells (significant level * for p < 0.05) at 0.5 uM CDP dose.

Patients received, according to the standard strategy of treatment for osteosarcoma [3] neoadjuvant chemotherapy that in this study consisted of two blocks of MTX (12 g/m^2^), CDP (120 mg/m^2^) and ADM (75 mg/m^2^) (Figure 1). MTX (12 mg/m^2^, top dose 24 g) was given i.v. over 4 hour (T0-T4) infusion. The minimum hydration required was 2.5 L/m2/day. From T24, folinc acid rescue was started every 6 hours for 11 administration (up to T84). CDP was given i.v. 120 mg/m2 over 48 hours. The minimum hydration required was 2 L/m^2^/24 hours After CDP infusion patients received ADM 75 mg/m^2^ i.v. over 24 hours.

All patients eligible for the study received the study drug ESOM. This was orally administered in the two days prior to each cytotoxic agent. Patients had to receive ESOM the two days before chemotherapy because preclinical studies have clearly shown that this is the only effective approach is the pretreatment [3]. The daily dose of ESOM was 60 mg/day (before MTX), that is the minimal effective dose calculated on the basis of the preclinical studies [3,4]. In patients weighting under 30 Kg the daily dose was reduced at 40 mg/day. Before CDP/ADM cycles, the daily dose of ESOM was 120 mg/day. The study drug was supplied by the local pharmacy.

Baseline studies included: complete blood count, serum electrolytes, and glomerular filtration rate estimation; serum alkaline phosphatase and lactate dehydrogenase levels; bilirubin and aminotransferase levels and echocardiography.

After neoadjuvant chemotherapy, the patients underwent surgical removal of the tumor.

Histological analysis of the tumor map was performed in accordance with a method reported previously [8]. When the percentage of tumor necrosis was equal or higher than 90%, patients were classified as good responders (GR); with a lower percentage they were defined as poor responders (PR).

Diagnosis, histological subtype, and histological response were reviewed by an expert panel of pathologists.

The rarity of osteosarcoma, with an incidence of 0.2/100,000 new cases/year [5], made it difficult to run a randomized phase 2 study, and for this reason it was decided to perform a prospective phase 2 study with a comparison with historical control.

The comparator of the present study was the previous Italian Sarcoma Group ISG/OS-1 study, whose results have already been published [14-17]. In particular, the group of patients treated according to arm A, that had received the same drugs and schedule adopted in the present study, was used as comparator.

In ISG/OS-1 the percentage of patients with a good pathologic response was 50%.

A sample size of 85 patients was calculated to detect a difference of 15% or higher, between the study group and the historical control (study power of 80%, type I error of 5%).

## Results

### Preclinical studies

#### In vitro

This set of experiments was aimed at defining the ability of the PPI ESOM to increase the sensitivity of human osteosarcoma cells to cytotoxic drugs. We first investigated the changes in the microenvironmental pH induced by the osteosarcoma cells growth. Osteosarcoma cells were able to acidify the medium by decreasing extracellular pH. In fact, after 24 h of cell culture in unbuffered medium, the extracellular pH (pHe) of both osteosarcoma cell lines (MG-63 and Saos-2) was respectively 6.67 ± 0.01 and 6.89 ± 0.05. Thus, there was the suitable condition for the best protonation of ESOM in order to allow the transformation of the pro-drug to the active molecule (tetracyclic sulfenamide).

Then we performed experiments aimed at evaluating the PPI-induced sensitization of human osteosarcoma cells to CDP. Results showed that 24 h ESOM pre-treatment significantly increased the activity of CDP in both osteosarcoma cell lines (Figure 1a and b). Particularly, after ESOM pre-treatment, the activity of CDP, at the lower dosage tested, was double in MG-63 and three times in Saos-2 model (p = 0.0277) compared to control. These results strongly supported the use of ESOM in the therapy of osteosarcoma patients.

#### In vivo

By analogy with the in vitro studies, we used the Saos-2 cellular model, ESOM as a PPI and CDP as one of the drugs currently used in treatment of osteosarcoma patients. The in vivo model was set up following the same procedure of the studies performed for other tumor types [3]. Briefly, CB.17 SCID/SCID mice were injected s.c. with 3 x10^6^ human osteosarcoma cells (Saos-2) and after 10 days 25 mg/kg ESOM was administered i.p., 24 hours before the i.p. injection of 5 mg/kg CDP. This treatment was repeated weekly for up to 4 consecutive weeks. Tumor size was measured three times/week until the end of the experiments. Three groups of at least 5 mice were treated with CDP alone, ESOM + CDP combination, or left untreated.

**Figure 2:**
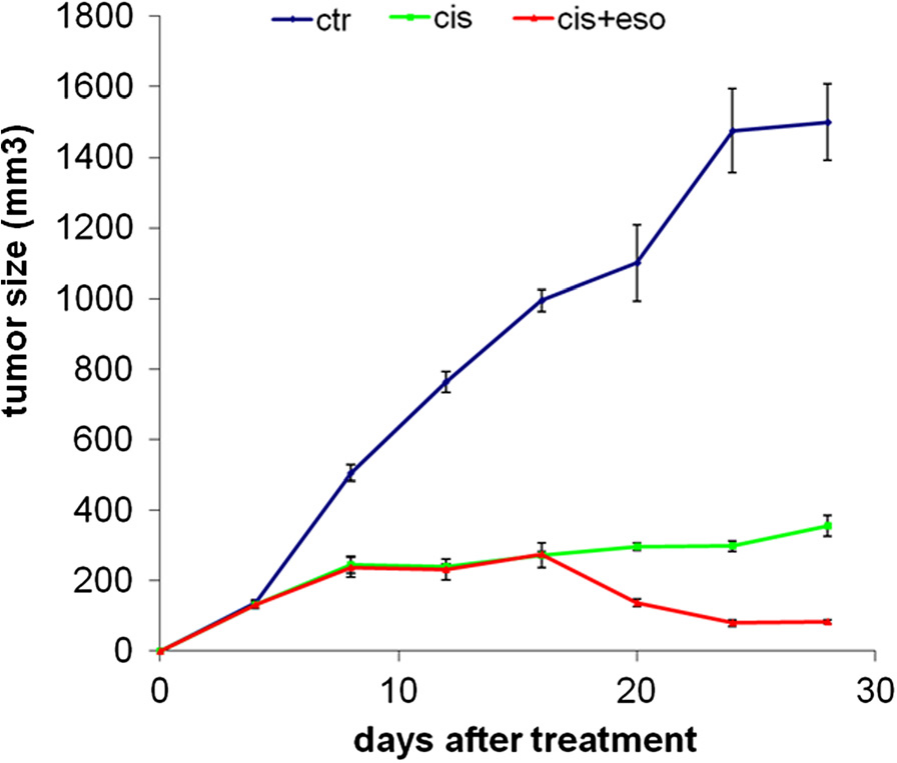
***In vivo *effects of ESOM on tumor growth in CB.17 SCID/SCID mice.** Mice were engrafted with Saos-2 cells via s.c. injection in the right flank. At the time of tumor appearance (approximately 7–10 days after injection), mice were left untreated or were pre-treated with 25 mg/kg of ESOM 24 hrs before the i.p. injection of CDP or treated with CDP alone. Tumor size was measured three times per week, and volume was calculated as described in the “Methods” section. The histograms represent mean +/− 95% confidence intervals of tumor weight.

Results showed that, as expected, CDP alone induced a significant inhibition of tumor growth, but also that ESOM pre-treatment induced a complete inhibition of tumor growth, with no evidence of the tumor at the end of the experiments (Figure 2). Supporting previous results obtained with human cell lines of different histotypes [3], ESOM alone, at this dosage and with this schedule of treatment did not induce significant inhibition of tumor growth (not shown). This set of experiments further supported the potential use of PPI in improving the effectiveness of chemotherapy in osteosarcoma patients.

### Clinical study

From January 2006 to December 2009, 99 patients [median age 15 years (6–40)] were registered into the study. One of them was excluded because a central revision did not confirm the diagnosis of osteoblastic osteosarcoma. All the 98 eligible patients were evaluable for histological response. No protocol deviation was registered. The clinical characteristics of eligible patients are summarized in Table 1.

**Table 1:** Clinical characteristics of eligible patients

**Variable**		**No (%)**
Gender	Male	59 (60%)
	Female	39 (40%)
Site	Femur	49 (50%)
	Tibia	33 (34%)
	Humerus	8 (8%)
	Other	8 (8%)
SAP	High	26 (26%)
	Normal	72 (74%)
LDH	High	22 (22%)
	Normal	76 (78%)
Histology	Osteoblastic	65 (66%)
	Chondroblastic	18 (18%)
	Telangiectatic	10 (10%)
	Fibroblastic	5 (5%)

**Figure 3:**
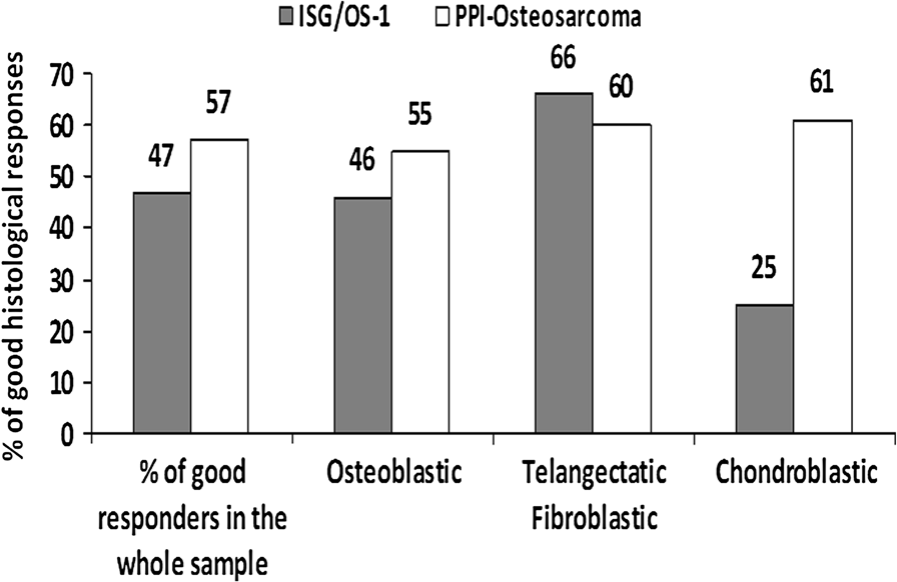
**Incidence of GR in eligible patients (PPI-Osteosarcoma) versus the comparator group (ISG/OS-1).** Comparison among the groups by means of Fisher’s exact test: Osteoblastic p = 0.32, Fibroblastic + Telangectatic 0.70, Chondroblastic p = 0.07.

A resection was performed in 93 patients, whereas 5 patients underwent amputation. The surgical margins evaluation was available in 93 patients. In 9 patients the surgical margins were classified as marginal (8 patients) or wide but contaminated (3 patients), the remaining patients had wide or radical surgical margins. On histology, a GR was detected in 56 (57%) patients (Figure 3). According to the histological histotype the highest rate of GR was seen in patients with the chondroblastic histotype followed by those with telangiectatic, fibroblastic, and osteoblastic variants (Figure 3). In the comparator group (ISG/OS-1), the incidence of GR was 47%, close to that observed in the investigational group (Figure 3). According to the histotype, a similar percentage of good response was observed in osteoblastic and in the pool of fibroblastic and telangiectatic osteosarcoma whereas in patients with chondroblastic osteosarcoma the incidence of GR was only 25%, a remarkably lower rate as compared to the 61% recorded in the group of patients pre-treated with ESOM.

Overall, the incidence of leukopenia G4 was 28%, thrombocytopenia G4 30%. The incidence of febrile neutropenia was 27%. Red blood cells and platelets transfusion were required in 18% and 16% of the chemotherapy cycles respectively. The incidence of delayed MTX excretion was 3% with transient G1 renal toxicity in 3 patients. Forty-two patients (mainly children and adolescents) experienced G4 transaminitis that in less than 30% of cases required medical treatment with steroids and fluid i.v. administration. In no cases the liver toxicity was cause of drug discontinuation, but it more frequently caused delay in the subsequent course of chemotherapy. No toxic deaths were reported. No serious adverse events were reported related to the pretreatment with ESOM.

The toxicity profile reported in the ISG/OS-1 study was similar to that observed in the PPI-Osteosarcoma study [14].

## Discussion

Microenvironmental acidity is a factor influencing the tumor homeostasis. Preclinical data suggest that a low pH is involved in resistance to cytotoxic drugs [3] and that tumor alkalinization through proton pump inhibitors (PPI), renders tumor cells more sensitive to chemotherapy [1,3].

Previous studies have shown as PPI have an anti-tumor effect against different tumor types [4,18], supporting the use of this family of compounds in the treatment of the vast majority of human tumors. Moreover, since 2010 the International Society for Proton Dynamics in Cancer (ISPDC), has gathered a consistent part of scientists that worldwide are studying tumor acidity and the proton dynamics underlying the low pH of tumors, with the aim to explore new modalities of treatment for cancer [19].

The preclinical experiments have shown that PPI pre-treatment induce a clear increase in the sensitivity of human osteosarcoma cells to chemotherapeutics. The *in vitro* experiments showed a significantly increase in the CDP-mediated cytotoxicity following ESOM pre-treatment against osteosarcoma cells. This set of experiments also provided the proof of concept that osteosarcoma cells cultured in unbuffered condition were fully able to acidify the medium, just providing the suitable condition for transformation of the ESOM pro-drug to the active molecule. In fact, in this condition while CDP alone was effective against MG-63 and Saos-2 osteosarcoma cells, esomeprazole significantly increased the CDP-mediated cytotoxicity at the lower dose of CDP. Notably, in MG-63 cells ESOM pre-treatment lowered ten times the dose of CDP able to induce a 50% inhibition of cell growth (0.5 μΜ vs 5 μΜ). The *in vitro* results were strongly supported by the in vivo results obtained in the human osteosarcoma-SCID mice xenografts. Consistently with the results obtained with the human osteosarcoma cell cultures, pre-treatment with ESOM significantly increased the effectiveness of CDP alone on the growth of human osteosarcoma in SCID mice, getting to a total inhibition of the tumor growth at the end of the experiments.

Thus, we tested the effectiveness of PPI pre-treatment in a clinical trial. The possibility to evaluate the pathological response to neoadjuvant chemotherapy by mapping the resected specimen and the clear relation between chemo-induced tumor necrosis and survival makes osteosarcoma an interesting model to assess a possible role of PPIs as an innovative therapeutic solution. The rarity of osteosarcoma made difficult to run a randomized phase 2 study, and for this reason we decided to perform a prospective phase 2 study with a comparison with historical control. On the other hand the expected pathological response to neoadjuvant chemotherapy in osteosarcoma is well defined, based on several previous experiences [16,20].

Overall, the addition of PPIs, to MTX, CDP, and ADM allowed a higher rate of GR compared to the control group, although this did not reach the statistical significance. It is important to notice that by comparing the histological response according to the different histotypes arises a difference for the chondroblastic variant, with a 61% of good responses in PPI pretreated patients (25% was the good response rate in the control group). It is well known that the expected rate of GR is low in the chondroblastic variant of osteosarcoma [20,21] and the results achieved in the present study suggest a relation between chondroblastic component, tumor acidity and the use of PPIs.

On the other hand it is important to underline that the results reported were achieved with a multidrug regimen, and that the drugs used have different pharmacokinetics and pharmacodynamics characteristic. For this reason the study does not allow any conclusions on the possible interaction between PPIs and each single drug used in the protocol.

The chemotherapy protocols currently used are characterized by a remarkable toxicity. Previous studies performed in the clinical centers involved in this project have shown that IV grade neutropenia is detectable in > 40% of patients despite the prophylactic treatment with G-CSF. The toxicity profile registered in patients who received pretreatment with ESOM was the same observed in patients having the same clinical characteristics and who received the same chemotherapy protocol. Preclinical data showing that PPI pre-treatment increases the sensitivity of human osteosarcoma cells to antineoplastic agents (particularly to CDP) is an interesting basis for further studies combining PPIs and antineoplastic agents at a reduced dose in an attempt to ameliorate the chemotherapy-related toxicity.

It is note worth, that a recent report highlighted the interaction between PPIs and MTX toxicity [22] leading to an increased MTX due to a concomitant use of the two drugs. In our report, however, the toxicity profile of MTX was not modified by pre-treatment with ESOM.

## Conclusions

This is the first translational study reporting preclinical and clinical investigations on the use of PPI in human osteosarcoma. Pre-clinical data clearly show that PPIs may directly affect the sensitivity of human osteosarcoma cells to chemotherapeutic drugs, such as CDP, and suggest that CDP could be delivered at reduced dose in an attempt to achieve a better toxicity profile.

Clinical data, while obtained with a surrogate endpoint, such as the chemotherapy-induced tumor necrosis, represent the first proof of concept that PPI may be included in the future strategies against cancer, and in particular in the treatment of osteosarcoma patients. The high pathological response rate in patients with the chondroblastic variant of osteosarcoma recommends further studies to assess the relation between PPIs and sarcoma tumors with chondroblastic component.

## Competing interests

The authors declare that they have no competing interests.

## Authors’ contributions

SF carried out the clinical study and drafted the manuscript. FP carried out the in vitro experiments and drafted the manuscript. FF participated in the clinical study. ABDP participated in the clinical study. CM participated in the clinical study AP participated in the clinical study. PP and MG evaluated the histological response in the clinical study SA participated in the in vitro experiments. NB drafted the manuscript. SF carried out the in vivo experiments, conceived of the study, and participated in its design and coordination and helped to draft the manuscript. All authors read and approved the final manuscript.
